# Case of Attempted Suicide, by Precipitation from Clifton Suspension Bridge

**Published:** 1885-06

**Authors:** J. Fenton Evans

**Affiliations:** House Surgeon, Bristol Royal Infirmary


### CASE OF ATTEMPTED SUICIDE, BY PRECIPITATION FROM CLIFTON SUSPENSION BRIDGE. By J. FENTON EVANS, M.B., House Surgeon, Bristol Royal Infirmary.

S. A. H., æt. 22 years, single, machinist, was admitted to Ward 16 on May 8th, at 2.0 p.m., having fallen from the Clifton Suspension Bridge about three-quarters of an hour previously. On admission the patient was exceedingly collapsed, pulseless at both wrists, face pale and anxious, lips livid. She could, however, be roused to give her name, and attempted to sit up, but sank back and nearly fainted. On being placed in bed, complained of pain in the chest, and of the extreme coldness of her limbs. Hot bottles and blankets were applied, and brandy swallowed without difficulty.

The condition of collapse, with weak pulse and constant shivering, persisted in a marked degree for about one hour after admission, the cardiac sounds being distinctly audible, but the heart's action weak and rapid. When the patient was sufficiently recovered to permit of more careful examination, the following injuries were made out:—Both lips slightly bruised; blood stains around mouth; separation of the first and second pieces of the sternum, with displacement forwards of first piece; tenderness and prominence over eighth and ninth right ribs, three inches in front of their angles; much bruising over both buttocks and back of thighs; feeble breathing over right lung as compared with left. No effusion of blood could be discovered at the nose, ears, mouth, or beneath the conjunctivæ. The pupils were equal, and acted to light somewhat sluggishly. No strabismus. No signs of the involuntary evacuation of the contents of the bladder or the bowel. At 4.0 p.m. respirations 36 per minute, pulse 120, weak. At 4.30 p.m. vomiting of fluid containing streaks of red blood.

May 9th. Patient has been delirious, and very restless all night. Axillary temperature 99·2, respirations 22, pulse 108, and stronger than yesterday. Limbs warm, colour in face and lips. No vomiting since yesterday afternoon, and no urine or motion passed since admission. Patient appears to-day to have fairly recovered from the shock, but talks incoherently from time to time, and has evidently no idea where she is, although in a ward with eleven other patients.

At 1 p.m. Twenty-four ounces of urine drawn off by catheter. Acid. sp., gr. 1017, albumen, $\frac{1}{6}$; on microscopic examination, found to contain numerous blood casts and blood corpuscles, white and red.

May 10th. Restlessness and delirium continue, although not so severe as yesterday. Vomiting during the night. This morning, pulse 96, respirations 28, temp.

98·4. Patient takes liquid nourishment readily. Voluntary micturition since yesterday: *urine*, acid. sp., gr. 1015; albumen, a trace; blood cells and blood casts present.

May 11th. Patient passed a very good night, sleeping for five or six hours. She is much more rational to-day, recognising her friends and those about her. Has asked who brought her to the Infirmary, and why; but has made no allusion to the real cause of her injuries. *Urine* to-day free from albumen and blood. Bowels moved for the first time since admission; no blood in fæces.

May 13th. Improvement continues.

May 27th. Patient is now quite convalescent, and able to walk without pain. There is apparently no permanent injury, beyond slight protrusion at the junction of the first and second pieces of the sternum. An examination of the tendon and skin reflexes reveals nothing abnormal.

*Remarks.*—The height of Clifton Suspension Bridge above the river at low water is about 250 feet, and the time any weight takes to fall that distance is about four seconds (leaving the resistance of wind, &c., out of the question). The time taken by this girl to fall from the bridge was probably much longer, not only on account of the resistance offered by the "rather high wind" blowing at the time, but also from the nature of her clothes, which are said to have been inflated from below, and thus delayed the descent. She was also reported to have fallen on the buttocks, which statement was supported by the condition of her clothes on admission, and the bruises subsequently discovered. A point of some interest is, that the patient's memory is now a complete blank as to what occurred after reaching the bridge, although she can remember setting out from home with the intention of destroying herself in this manner. Of the sixteen persons who have hitherto attempted suicide from this bridge, this girl alone has survived the consequences of the act; one other was picked up alive, and survived twenty or thirty minutes. As far as the writer knows, no case of survival after a fall from a height of 150 feet has hitherto been recorded; this instance of recovery, after a fall of nearly 250 feet, is probably unique.

---

